# Genome mining conformance to metabolite profile of* Bacillus* strains to control potato pathogens

**DOI:** 10.1038/s41598-023-46672-1

**Published:** 2023-11-04

**Authors:** Arezoo Lagzian, Roohallah Saberi Riseh, Sajjad Sarikhan, Abozar Ghorbani, Pejman Khodaygan, Rainer Borriss, Pietro Hiram Guzzi, Pierangelo Veltri

**Affiliations:** 1 Department of Plant Protection, Faculty of Agriculture, Vali-e-Asr University of Rafsanjan, Rafsanjan, Iran; 2 Molecular Bank, Iranian Biological Resource Center (IBRC), ACECR, Tehran, Iran; 3 Nuclear Agriculture Research School, Nuclear Science and Technology Research Institute, Karaj, Iran; 4 Institute of Biology, Humboldt University Berlin, Berlin, Germany; 5 Department of Surgical and Medical Sciences, University of Catanzaro, Catanzaro, Italy; 6 Department of Informatics Modeling Electronics and System Engineering, University of Calabria, Calabria, Italy

**Keywords:** Microbiology, Molecular biology, Plant biotechnology

## Abstract

Biocontrol agents are safe and effective methods for controlling plant disease pathogens, such as *Fusarium solani*, which causes dry wilt, and *Pectobacterium* spp., responsible for potato soft rot disease. Discovering agents that can effectively control both fungal and bacterial pathogens in potatoes has always presented a challenge. Biological controls were investigated using 500 bacterial strains isolated from rhizospheric microbial communities, along with two promising biocontrol strains: *Pseudomonas* (T17-4 and VUPf5). *Bacillus velezensis* (Q12 and US1) and *Pseudomonas chlororaphis* VUPf5 exhibited the highest inhibition of fungal growth and pathogenicity in both laboratory (48%, 48%, 38%) and greenhouse (100%, 85%, 90%) settings. Q12 demonstrated better control against bacterial pathogens in vivo (approximately 50%). Whole-genome sequencing of Q12 and US1 revealed a genome size of approximately 4.1 Mb. Q12 had 4413 gene IDs and 4300 coding sequences, while US1 had 4369 gene IDs and 4255 coding sequences. Q12 exhibited a higher number of genes classified under functional subcategories related to stress response, cell wall, capsule, levansucrase synthesis, and polysaccharide metabolism. Both Q12 and US1 contained eleven secondary metabolite gene clusters as identified by the antiSMASH and RAST servers. Notably, Q12 possessed the antibacterial locillomycin and iturin A gene clusters, which were absent in US1. This genetic information suggests that Q12 may have a more pronounced control over bacterial pathogens compared to US1. Metabolic profiling of the superior strains, as determined by LC/MS/MS, validated our genetic findings. The investigated strains produced compounds such as iturin A, bacillomycin D, surfactin, fengycin, phenazine derivatives, etc. These compounds reduced spore production and caused deformation of the hyphae in *F. solani*. In contrast, *B. velezensis* UR1, which lacked the production of surfactin, fengycin, and iturin, did not affect these structures and failed to inhibit the growth of any pathogens. Our findings suggest that locillomycin and iturin A may contribute to the enhanced control of bacterial pectolytic rot by Q12.

## Introduction

Fungal pathogens, such as *Fusarium solani* (FS)^1,2^, and bacterial pathogens, including *Pectobacterium carotovorum* (PC)^3,4^, *P. atrosepticum* (PA)^5–8^, and *Xanthomonas campestris* (XC)^9^, play a significant role in global plant diseases, causing root rot (dry and soft), leaf spots, and blackleg in both forest and crop ecosystems^1,7,10^. The use of chemical fungicides and bactericides for disease management has adverse effects on human health and the environment. Consequently, there is an increasing reliance on biological agents to mitigate the negative impact of agrochemicals. The rhizosphere represents a competitive environment where microorganisms vie for resources to ensure their survival^11^. While beneficial microorganisms are widely used in agriculture to control plant diseases, their inconsistent performance and a lack of sufficient safety data have limited the adoption of many promising biopesticides^12^. Thus, there is an urgent need to identify bacteria that not only promote plant growth but also consistently demonstrate effective biological control mechanisms. Genome mining of microorganisms can provide valuable insights into the molecular-level mechanisms involved. Plant growth-promoting rhizobacteria (PGPR) employ various mechanisms for biocontrol. In addition to secondary metabolites that directly act against plant pathogens^13^, induced systemic resistance (ISR) and rhizospheric colonization are key mechanisms for competing with pathogens^14^. PGPR can engage with pathogenic agents through parasitism or antibiosis, wherein they counteract pathogen attacks and disrupt hyphae, spores, and bacterial cell networks^15^. Lipopeptides (LPs) are produced by diverse groups of both gram-positive and gram-negative bacteria, fungi, and yeasts. The wide variety of LP-producing microorganisms and the differences in their chemical structures suggest that cyclic and linear LPs may serve multiple purposes. In *Bacillus* and *Pseudomonas*, LPs play essential roles in stimulating plant-induced systemic resistance (ISR), antagonism, motility, and surface attachment^13^. *Bacillus* spp. and *Pseudomonas* spp*.* are currently employed as biocontrol agents to mitigate damage caused by bacterial and fungal pathogens, constituting more than half of commercial biopesticides. In addition to ribosomally synthesized antimicrobial peptides (bacteriocins), *Bacillus* spp. can synthesize numerous lipopeptides through nonribosomal peptide synthetases (NRPS), displaying broad-range biological activities^16,17^.

In this study, our objectives were threefold: firstly, to identify the most effective strains among 500 bacterial isolates for controlling the four selected plant pathogens; secondly, to investigate the genetic structure of the entire genome in order to identify the biosynthetic gene clusters responsible for secondary metabolite production in these strains; and thirdly, to monitor the expression of secondary metabolites in the bacteria and study their roles in disease prevention.

## Materials and methods

### Microorganisms and pathogens

Biocontrol bacteria were isolated from rhizosphere soil samples obtained from healthy potato plantations. We collected 500 bacterial strains from diverse rhizosphere microbial communities and cultured them on nutrient agar plates at varying dilutions. In previous studies, we isolated two strains, VUPf5 and T17-4, from a pool of nine hundred bacterial isolates originating from different regions of the country. VUPf5 exhibited superior biocontrol properties against various plant pathogens in both in vitro and in vivo settings, while T17-4 demonstrated excellent performance in laboratory tests as well^18–21^. In this study, we aimed to identify these two strains, further investigate their effective characteristics, and employ VUPf5 and T17-4 as positive biocontrol treatments to identify better functional comparative agents for controlling potato diseases. Pathogenic bacterial strains of *Pectobacterium carotovorum*, *P. atrosepticum,* and *X. campestris* were sourced from the Biotechnology and Biomedicine Department collection at the Technical University of Denmark. The pathogenic fungus *F. solani* was obtained from the Agricultural and Natural Resources Research Center in Iran. Strains *B. velezensis* Q12 and US1 were deposited in the Collection of Biological Control of Plant Pathogens at the University of Vali-e-Asr.

### Anti-*Fusarium* effects under planktonic conditions

To assess the anti-*Fusarium* effects of biocontrol bacteria, bacterial suspensions were cultivated to OD_600_ 0.2 and applied to potato dextrose agar (PDA) plates, positioned one cm from the Petri dish edge. *Fusarium* mycelium plugs (1 cm diagonal) were placed in the center of the plates, and incubation was carried out at 25 °C^22^. The inhibition zone was measured as the distance between the growth margins of mycelia and bacteria when *Fusarium* covered the entire control plate. Plates were examined under a stereomicroscope, and strains with optimal, moderate, and poor inhibition abilities were further screened in the greenhouse and tested against pathogenic bacteria. Each treatment consisted of three replicates, and experiments were repeated three times.

### Anti-microbial effects under planktonic conditions

To investigate the antibacterial effects of biocontrol bacterial strains against the plant pathogens *P. carotovorum*, *P. atrosepticum*, and *X. campestris,* we used half-strength lysogeny broth agar medium in Petri dishes. Pathogenic bacteria were introduced into the medium to reach a final concentration of OD_600_ 0.2. Subsequently, cultures (OD_600_ 0.2) of antagonistic bacteria (5 µl) were added to the plates. The plates were incubated at 28 °C for 72 h, and the halo diameter around bacterial colonies was examined daily. Stereomicroscopy was used for plate investigation and imaging. Each treatment included three replications.

### Application of micro-tuber coating for *Fusarium* Wilt

To evaluate the efficacy of micro-tuber coating against *Fusarium* wilt, disinfected micro-tubers were treated with biocontrol bacteria (10^9^ CFU/ml) after rinsing with sterile distilled water. Soil in pots was inoculated with 2 × 10^4^ spores g^−1^ substrate^23^. The planted potato micro-tubers were exposed to contaminated soil. Pots containing only pathogenic fungi served as the positive control, while pots without the pathogen and antagonists constituted the negative control. The pots were placed in a greenhouse at 23–25 °C and irrigated every three days. After 45 days, leaf and root symptoms were assessed for disease extent. Disease Index percentages (formulas 1 and 2) and root and total plant weights were measured for each treatment. Each treatment was replicated three times in a completely randomized design^24^. Statistical analyses were conducted using One-Way ANOVA and the Post Hoc Test (Duncan test) at a significance level of 0.01.

- X: Disease scale between 0–8
- $$ {\text{N1}}:{\text{ Total number of seedlings planted}}\;\% {\text{DI}} = [\mathop \sum \limits_{k = 1}^{n} \left( {{\text{N}}2 \times {\text{X}}} \right) \div \left( {{\text{N}}1 \times 8} \right)] \times 100 $$
- N2: Number of seedlings planted in a treatment.
- Disease reduction in treatment% = Disease severity in infected control%- Disease index in treatment% (2).

### Biocontrol strains against bacterial pathogens on potato tubers

Biocontrol bacterial strains were propagated using the spotting method on plates. Subsequently, 5 µl of each strain was injected into potato tubers under sterile conditions, and an equal volume of each bacterial pathogen (*P. carotovorum* or *P. atrosepticum*), (OD_600_ 0.2), was added to the created well and sealed with sterile glue. Bacterial pathogen spreading diameter was evaluated after 72 h of incubation at 28 °C. Each treatment was replicated three times, and the experiment was repeated twice.

### DNA extraction and amplification of genes and sequencing

Seven rhizobacterial strains were selected for taxonomical assignment. These strains were cultured aerobically at 28 °C with continuous shaking at 220 rpm in rich lysogeny broth (Lenox LB, Carl Roth, Germany). Cells were precipitated by centrifugation at 10,000 rpm, and DNA was extracted using the EUR_x_® Bacterial Genomic DNA Purification Kit. DNA concentration was determined using Nanodrop. In this study, six pairs of primers were used for bacterial identification. Primers Fw_27F (5’-AGAGTTTGATCMTGGCTCAG-3’) and Rv_1492R (5’-TACGGYTACCTTGTTACGACTT-3’) were employed to amplify the 16S rRNA regions in all strains. The *rpoD* primers, Fw_PsEG30F (5’-ATYGAAATCGCCAARCG-3’) and Rv_PsEG790R (5’-CGGTTGATKTCCTTGA-3’) were used for gram-negative strains. Additionally, the genetic sequence of the *gyrA* gene was amplified using Fw_42F (5’-CAGTCAGGAAATGCGTACGTCCTT-3’) and Rv_1066R (5’-CAAGGTAATGCTCCAGGCATTGCT-3’) primers for gram-positive bacteria. PCR reactions were conducted with final concentrations of 1X PCR Buffer, 2.5 mM MgCl2, 0.6 mM dNTPs, 0.4 μM of each primer, and 2 Units of Taq DNA polymerase in a final volume of 50 μl. The PCR conditions included 95 °C for 5 min, followed by 30 cycles of 95 °C for 45 s, 54 °C, 58 °C, and 50 °C for 16S rRNA, *gyrA*, and *rpoD* primers, respectively, for 30 s. The PCR continued at 72 °C for 30 s and concluded at 72 °C for 10 min. PCR products were visualized on a 1% agarose gel under UV light and then purified using the NucleoSpin® Gel and PCR cleanup kit. Sequence data were analyzed and identified using CLC Genomic Workbench software version 20.0.

### UHPLC-HRESIMS analysis

For this experiment, agar plugs (PGA and LBA) containing bacterial cultures (Q12, US1, VUPf5, and T17-4) and a control treatment without any bacterial culture were transferred to a vial after three days of incubation at 25 °C. The plugs were dissolved in 1 mL of ethyl acetate under ultrasonication for 60 min. The solutions were transferred to new vials, dried, evaporated by N_2_, and re-suspended in 200 µL of methanol. Samples were further subjected to sonication or vortexing for 15 min before centrifugation at 13,400 rpm for three min. Supernatants were transferred to vials and subjected to ultrahigh-performance liquid chromatography-high resolution electrospray ionization mass spectrometry (UHPLC-HRESIMS) analysis using an Agilent Infinity 1290 UHPLC system equipped with a diode array detector. HPLC was conducted using a phenyl-hexyl column at 60 °C with a mobile phase consisting of acetonitrile (ACN) and water containing 20 mM formic acid (FA). A linear gradient of ACN/H_2_O to ACN (10% to 100%) for 10 min was applied, followed by isocratic elution of 100% ACN for 2 min. Samples were then adjusted to 10% ACN/H_2_O for 0.1 min, and eventually, the isocratic state of 10% ACN/H_2_O was reached in 1.9 min. The flow rate was set at 0.35 mL/min. Mass detection was performed using an Agilent 6545 QTOF MASS equipped with an Agilent Dual Jet Stream electrospray ion source. Positive ionization was employed, and instrument parameters were set as follows: drying gas temperature (250 °C), drying gas stream (8 L/min), sheath gas temperature (300 °C), sheath gas stream (12 L/min), capillary voltage (4000 V), and nozzle voltage (500 V). Mass data analysis was carried out using Agilent MassHunter Qualitative Analysis B.07.00. Each treatment included three replicates, and experiments were conducted three times.

### Next-generation sequencing and analysis

For whole-genome sequencing, bacterial strains Q12 and US1 were cultivated to an OD_600_ of 0.25–0.5. After centrifugation, the pellets were resuspended in 500 μl of cryopreservation solution (Microbank™, Pro-Lab Diagnostics UK, United Kingdom). Approximately 2 × 10^9 cells were used for DNA extraction, which was carried out using the Nanobind CCB Big DNA Kit. Genome sequencing was performed using both the Illumina HiSeq and the Nanopore platform. DNA libraries were prepared with the Nextera XT Library Prep Kit and sequenced on the Illumina HiSeq with a 250 bp pair. Trimmomatic 0.30^25^ was used to trim Illumina reads, and long-read libraries were obtained with the Oxford Nanopore SQK-RBK004 kit. Genome assembly was conducted using Unicycler v0.4.0^25,26^, and circular contigs were annotated with Prokka 1.11^27^, RAST PATRIC, antiSMASH (6.0), and DAVID servers. Gene ontology and pathways analyses were performed using DAVID servers for each bacterium. Sequence data have been deposited in NCBI with accession numbers: CP076290 (*Bacillus velezensis* Q12), CP076291 (*B. velezensis* US1 chromosome), and CP076292 (*B. velezensis* US1 plasmid). Each experiment was performed in triplicate, and results were validated in three independent experiments.

### Ethics approval

This article does not contain any studies with human participants or animals performed by any of the authors.

### Consent to participate

There were no human participants so there was no need for their consent to participate.

## Results

### Anti-*Fusarium* effects under planktonic conditions

**Figure 1:**
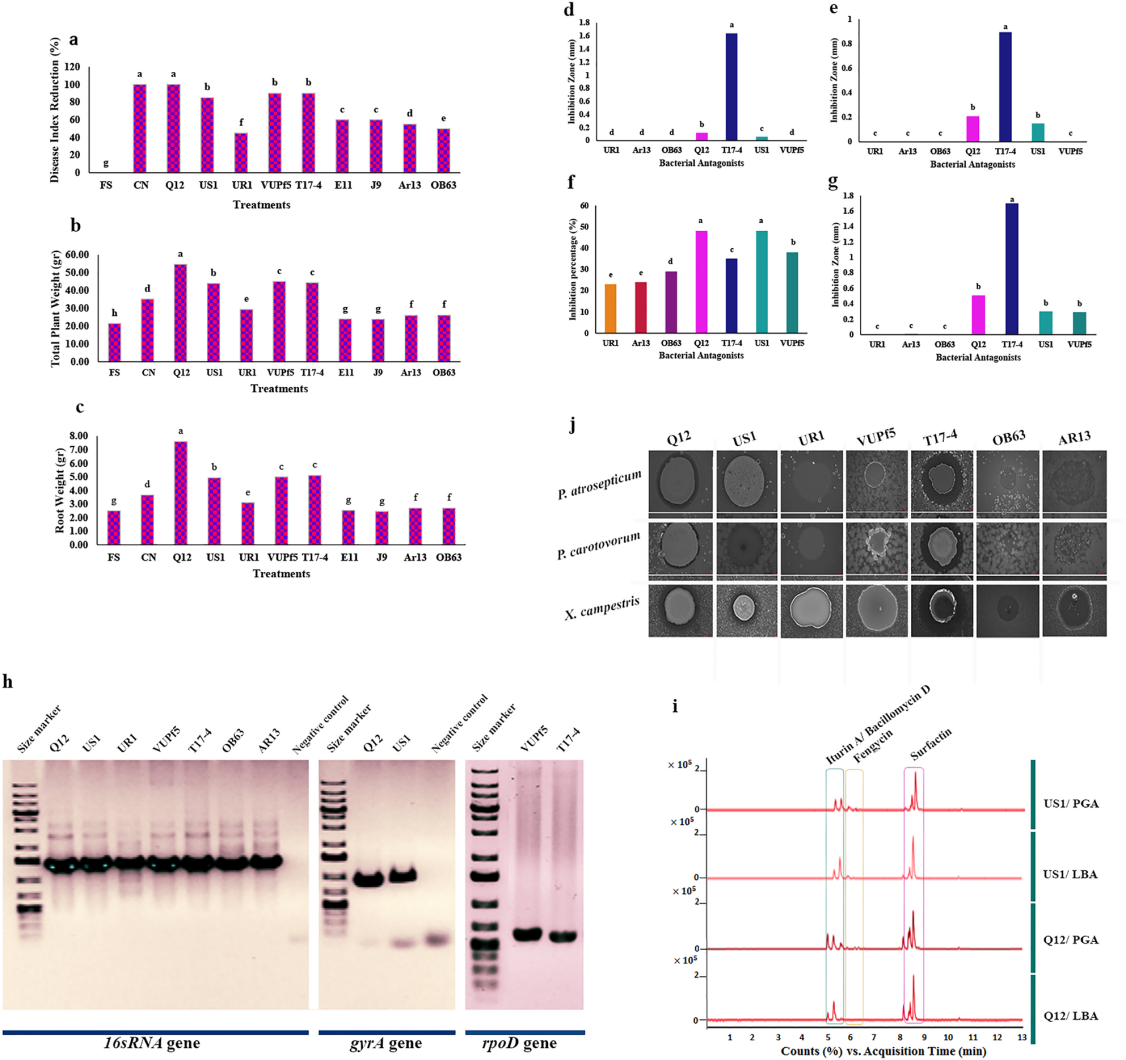
(**a**–**c**). Analysis of greenhouse experiment using One-Way ANOVA, *p* < 0.01. DIR (%), Percentage of disease index reduction. CN, control negative without any pathogen or biocontrol bacteria. (**d**–**g**). Interaction between biocontrol bacteria, bacterial and fungal pathogens. (**d**). *P. carotovorum*, (**e**). *P. atrosepticum*. (**f**). *F. solani*. (**g**). *X. campestris*. Analysis was performed by One-Way ANOVA, *p* < 0.01. (**h**). Molecular identification of bacterial strains. Size Marker (1 Kb plus, Thermo Fisher). Amplified by 16sRNA primers (27F and 1492R), *gyrA* primers, and *rpoD* primers (left to right). (**i**). Investigating the production of secondary metabolites and showing chromatograms using LC/MS/MS. Peaks of production of Bacillomycin D, Fengycin and Surfactin in *B. velezensis* US1 on PGA and LBA and peaks of production of Iturin A, Fengycin and Surfactin in *B. velezensis* Q12 on PGA and LBA, respectively. (**j**). Interaction between biocontrol bacteria and bacterial pathogens is indicated by the inhibition zones around the antagonists.

Out of approximately 500 isolates, five strains demonstrated efficient suppression of the plant pathogen *F. solani *in vitro, with 20% of all isolates displaying moderate control ability against it. After initial screening, a total of seven strains exhibiting high or weak antagonistic activity were selected for further experiments. These selected strains underwent taxonomic assignment through sequencing of the 16S rRNA, *rpoD*, and *gyrA* genes. Subsequently, the sequences were analyzed using CLC Genomic Workbench software version 20.0 and deposited in the BanKit/NCBI and GenBank/NCBI databases. The strains Q12 (accession numbers MZ357346 and MZ310706), US1 (accession numbers MZ357347 and MZ310808), and UR1 (accession numbers MZ357348 and MZ310821) were identified as *B. velezensis*, while the strains VUPf5 (accession numbers MZ346009 and MZ349056) and T17-4 (accession number MZ346008) were identified as *P. chlororaphis* and *P. aeruginosa*, respectively. Additionally, OB63 (accession number MZ312100) and AR13 (accession number MZ310814) were classified as *Escherichia coli* (Fig. 1h).

The impact of these rhizospheric bacteria on the growth rate of the plant pathogen was assessed on PGA media. The presence of these bacterial strains significantly influenced the reduction of fungal growth (F = 324.00, df = 6, *p* = 0.000). *B. velezensis* Q12 and *B*. velezensis US1 exhibited the highest inhibition rates (48%) against *F. solani*. *P. chlororaphis* VUPf5 (38%), *P. aeruginosa* T17-4 (35%), *E*. *coli* OB63 (29%), *E*. *coli* Ar13 (24%), and *B*. *velezensis* UR1 (23%) displayed varying degrees of growth inhibition. After seven days of treatment with *P. chlororaphis* VUPf5 and *P. aeruginosa* T17-4, spore formation of *F. solani* was significantly reduced. Notably, VUPf5 also disrupted the mycelium network, resulting in fragmented fungal filaments around the bacterial cells. Complete cessation of hyphal growth and reduced sporulation were observed in the presence of B. velezensis Q12. Conversely, treatment with *B*. *velezensis* US1 did not lead to a significant change in the number of fungal spores, and *B*. *velezensis* UR1 showed ineffective control of *F. solani*. The two strains of *E*. *coli* did not efficiently control *F. solani*, as evidenced by a large number of fungal spores surrounding the bacterial cells (Fig. 1f; Fig. S1; Fig. S2; Fig. S3, Supplementary Table 1).

### Anti-microbial effects under planktonic conditions

The selected strains were further evaluated for their effects against the bacterial pathogens *Pectobacterium* spp. (PC and PA) and *Xanthomonas campestris* (XC) under planktonic conditions. One-way ANOVA analysis revealed significant differences in bacterial prevention among the co-culture treatments with biocontrol bacteria. Post-hoc tests indicated significant differences between the treatments in the control groups of PC (F = 3128.16, df = 6, *p* = 0.000), PA (F = 143.33, df = 6, *p* = 0.000), and XC (F = 57.07, df = 6, *p* = 0.000). The highest rate of bacterial growth inhibition on LB agar was observed with *Pseudomonas aeruginosa* T17-4 and *B. velezensis* Q12. In contrast, *B*. *velezensis* US1 was less effective in controlling *P. atrosepticum* and *X. campestris* (Fig.1d,e,g,j; Supplementary Table 1). However, these in vitro results do not necessarily imply that *P. aeruginosa* T17-4 is superior to other biocontrol agents against bacterial pathogens under in vivo conditions. Co-culturing potato tubers with T17-4, PC, and PA showed higher susceptibility to the pathogens compared to control experiments with biocontrol bacilli.

### Application of micro-tuber coating for *Fusarium* Wilt

Nine strains were selected to evaluate their effect on *F. solani* (FS) under in vivo conditions. Inoculation with FS resulted in significantly different levels of root rot disease severity among the tested plants (F = 52,576.41, df = 10, *p* = 0.001). Treatment with UR1 showed the lowest effect on the disease and resulted in the highest overall disease severity rating (DSR) with a mean disease index reduction (DIR) of 45%. This was followed by treatments with AR13 (DIR = 54.5%), OB63 (DIR = 53.5%), E11 (DIR = 60%), and J9 (DIR = 60%). In contrast, inoculation with Q12*,* US1, T17-4, and VUPf5 yielded lower overall disease symptoms and a disease index reduction of 100%, 99%, 99%, and 99%, respectively. Symptoms of fungal infection were not observed in Q12, which also had the highest root and total plant weight (g) (Fig. 1a–c). Post-hoc tests indicated significant differences between the treatments in root weight (F = 713.78, df = 10, *p* = 0.001) and total plant weight (F = 1642.26, df = 10, *p* = 0.001).

### Genome sequencing analysis

The RAST and PATRIC annotated genomes of two strains, *B. velezensis* US1 and Q12, contained 4,132,553 bps, 4, 396 genes, and 4,255 coding sequences with a GC content of 46.0%, and 4,182,261 bps, 4, 443 genes, and 4,300 coding sequences with a GC content of 46.1%, respectively. The annotated unique genes in the two strains exhibited great similarity in some subsystems and the number of genes. However, 45 specific gene IDs were identified in Q12 that were not present in US1. Differences were observed in the number of relevant genes related to stress response, cell wall, and capsule.

**Figure 2:**
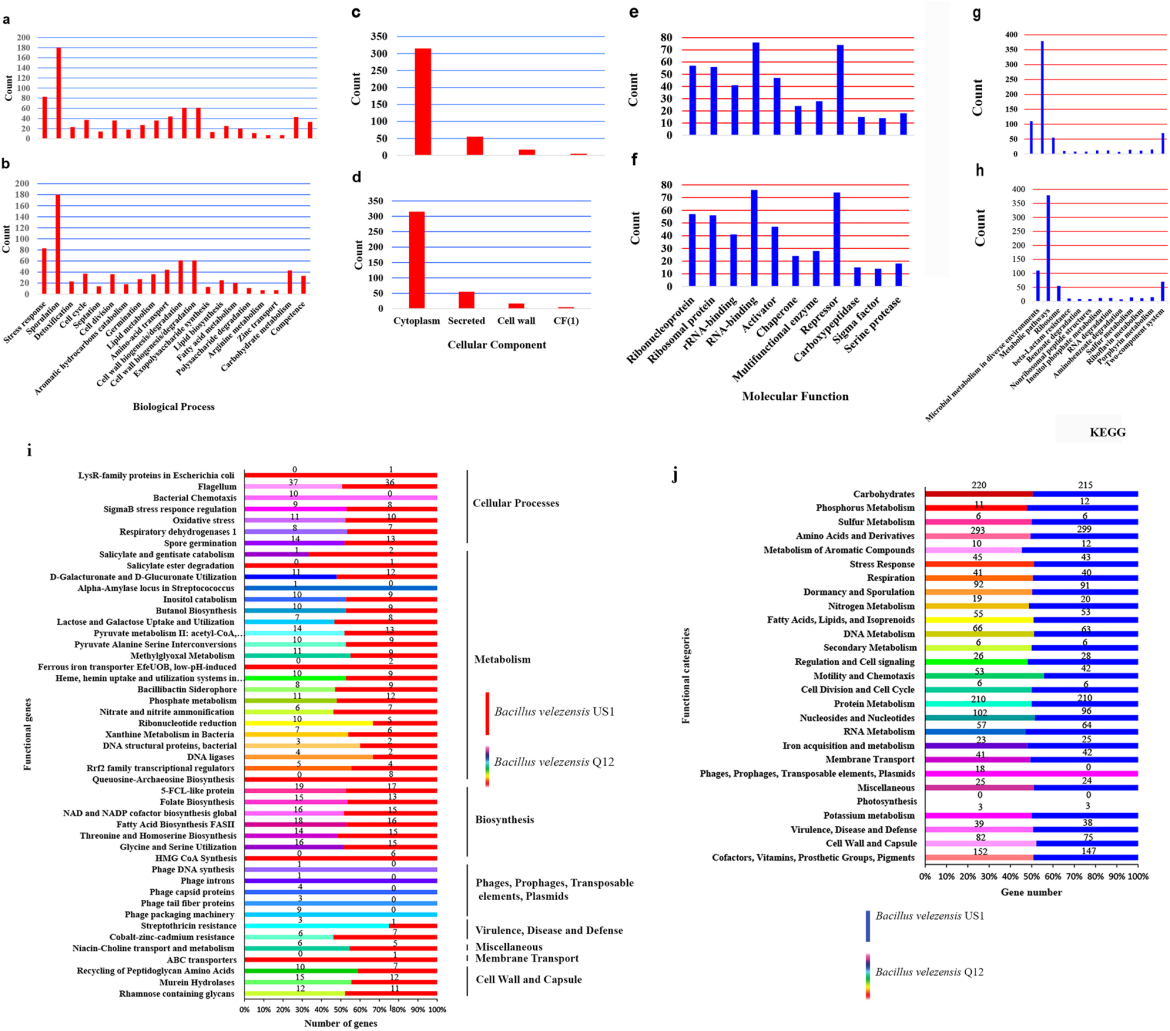
(**a**–**j**) Gene Ontology (GO) in *B. velezensis* Q12 and *B. velezensis* US1. (**a**–**h**). Annotation analyses were performed by the DAVID server and in (**i**) and (**j**) by the RAST server.

**Figure 3:**
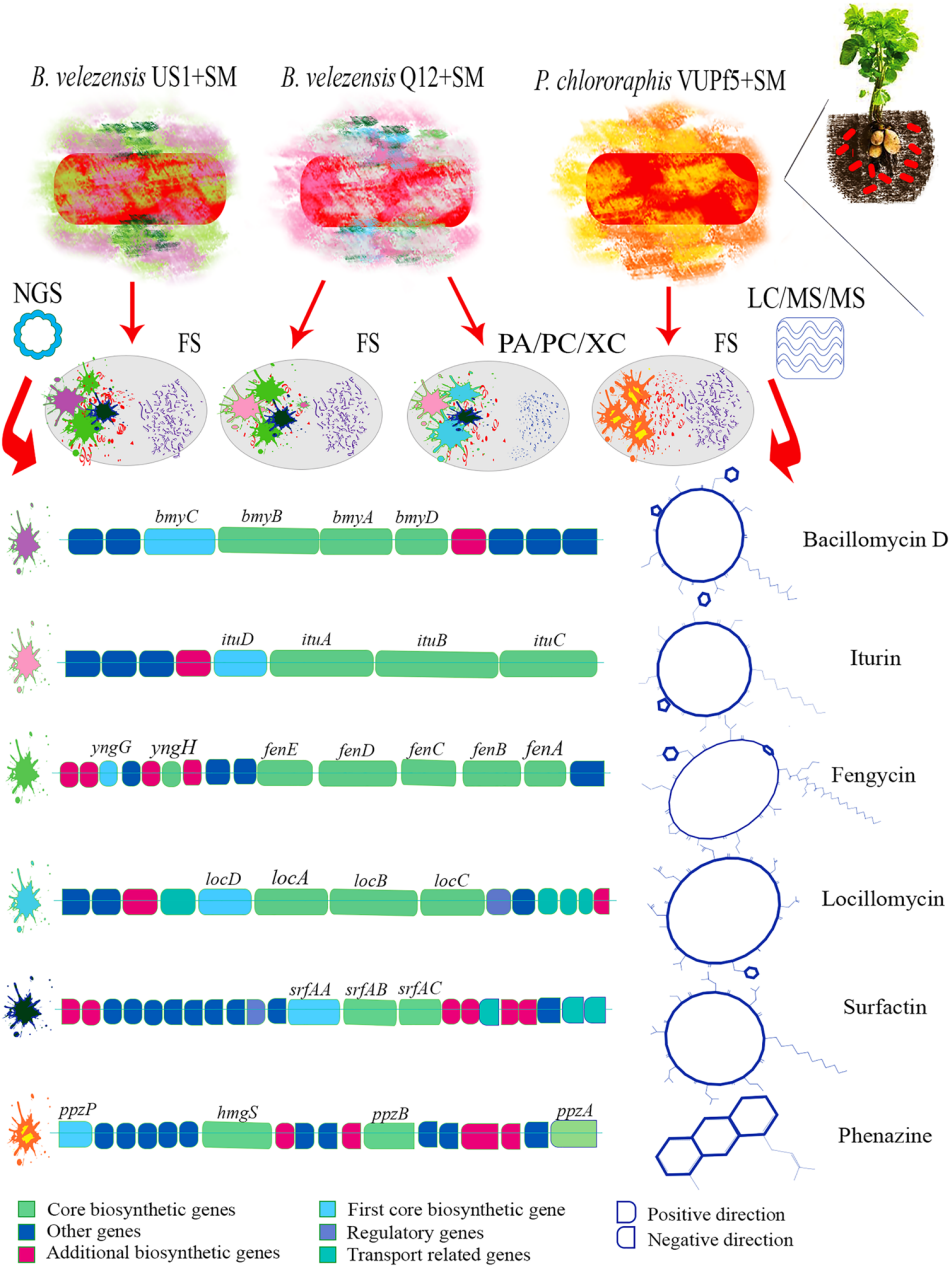
Gene clusters and secondary metabolites structures in *B. velezensis* Q12, *B. velezensis* US1, and *P. chlororaphis* VUPf5. SM: secondary metabolites.

For example, strain Q12 had two more genes (45) compared to US1 (43) in the oxidative stress response gene (Organic hydroperoxide resistance protein) and sigmaB stress response regulation (RsbR, positive regulator of sigmaB). Furthermore, approximately seven more genes appeared within the cell wall and capsule subcategory in strain Q12. One rhamnose-containing glycans gene, which is classified in the subsystem of capsular and extracellular polysaccharides, three genes of murein hydrolases, and three other genes from the recycling of peptidoglycan amino acids subsystem were found in Q12, but not in US1. Polysaccharide metabolism and levensucrose biosynthesis gene (*sacB*, from 4131008 to 4132429 on the positive strand) were found in Q12 but were absent in US1 (Fig. 2a–j). In other categories, slight differences were observed in some genes. The auxin plant hormone genes were identical in both strains, including four genes: tryptophan synthase alpha chain, anthranilate phosphoribosyltransferase, tryptophan synthase beta chain, and phosphoribosyl anthranilate isomerase. The antiSMASH server was used to annotate and group the bacterial genomes into 25 and 27 cellular functional metabolic regions for US1 and Q12, respectively, which included eleven secondary metabolite gene clusters (surfactin, plantazolicin, butirosin A/B, macrolactin H, bacillaene, fengycin, bacillomycin D, difficidin, bacillibactin, teichuronic acid, bacilysin) and (locillomycin, surfactin, butirosin A/B, macrolactin H, bacillaene, fengycin, Iturin A, difficidin, bacillibactin, teichuronic acid, bacilysin) in US1 and Q12, respectively. The SM gene clusters were found in the non-ribosomal peptide (NRP), polyketide (PK), NRP + PK, and saccharide regions (Fig. 3). The Gene Ontology (GO) is an important bioinformatics tool that provides an international standard description for the biological, structural, and molecular function of unique genes. GO has three perspectives, namely, biological process (BP), cellular component (CC), and molecular function (MF), with 20, 4, and 11 subfunctions, respectively. In this study, 779, 392, and 450 gene IDs were obtained from BP, CC, and MF, respectively. The Kyoto Encyclopedia of Genes and Genomes (KEGG) was also used to define the genome delineation for possible metabolic processes or functions, revealing 13 metabolic pathways in both Q12 and US1. Among the identified genes, eight were related to non-ribosomal peptide structures, specifically non-ribosomal plipastatin (*ppsA*, *ppsB*, *ppsC*, *ppsD*, and *ppsE*) and surfactin (*srfAA*, *srfAB*, and *srfAC*) synthetase genes. The plipastatin polypeptide belonged to the fengycin family. Protein and nucleotide Blast analysis of the antiSMASH database showed that the mentioned genes had 100% similarity to the fengycin gene cluster in both strains and revealed 84% similarity to the plipastatin gene cluster (Fig. 2a–h).

### LC/MS/MS assay

An LC/MS/MS assay was performed to confirm the expression of several secondary metabolites encoded by the genome. The results from LC/MS/MS and annotation analysis indicated that both strains produced secondary metabolites with antifungal and antibacterial activity, as well as siderophores. Strain Q12 produced surfactin with a retention time (RT) of 9.2 min, fengycin with an RT of 6.5 min, and iturin A with an RT of 5.5 min (Table 1; Fig. 1i). Strain US1 produced surfactin with an RT of 9.2 min, fengycin with an RT of 6.5 min, and bacillomycin D with an RT of 5.8 min. However, strain UR1, which was unable to develop antimicrobial effects, did not produce significant amounts of surfactin, fengycin, and iturin (Fig. S4). Additionally, the *Pseudomonas* strains expressed phenazines. Derivatives of phenazine-1-carboxylic acid (PCA) with an RT of 5.5, 2-hydroxyphenazine-1-carboxylic acid with an RT of 5.6, and 2-hydroxyphenazine with an RT of 4.3 were produced in VUPf5. Strain T17-4 also produced 2-Heptyl-3-hydroxy-4-quinolone (PQS) with an RT of 5.8 and rhamnolipid A with an RT of 8.

**Table 1:** Secondary metabolites production in rhizospheric bacteria based on whole genome sequencing and ultrahigh-performance liquid chromatography; high-resolution electrospray ionization mass spectrometry.

Bacteria	Media	Secondary Metabolite
*B. velezensis* Q12	PGA/LBA	iturin A*- fengycin*- surfactin*- locillomycin- butirosin A/B- macrolactin H- bacillaene- difficidin- bacillibactin- bacilysin
*B. velezensis* US1	PGA/LBA	bacillomycin D* -fengycin*—surfactin* -plantazolicin- butirosin A/B- macrolactin H- bacillaene- difficidin- bacillibactin- bacilysin
*P.* *chlororaphis* VUPf5	PGA/LBA	phenazine-1-carboxylic acid*—2-hydroxyphenazine-1-carboxylic acid*—2-hydroxyphenazine*—lahorenoic acids A*
*P.* *aeruginosa* T17-4	PGA/LBA	phenazine-1-carboxylic acid*- 2-hydroxyphenazine*- pyochelin*- PQS*- Rhamnolipid *—phenazine-1-carboxamide*
*B. velezensis* UR1	PGA/LBA	expression of iturin- fengycin- surfactin not enough to trace*

*They were also detected by UHPLC-HREIMS.

## Discussion

### Biocontrol of *F. solani* in the laboratory

This study demonstrated the biocontrol potential of selected strains of *B. velezensis* and two previously investigated strains, *P. chlororaphis* VUPf5 and *P. aeruginosa* T17-4, against fungal dry wilt and bacterial soft rot of potatoes. Notably, strain VUPf5 exhibited a profound impact on *F. solani*, leading to the fragmentation of fungal filaments around bacterial cells. This effect could be attributed to the production of lytic enzymes, such as chitinase and laminarinase, which hinder mycelial development and cause hyphal lysis in *F. solani*^28^.

*Pseudomonas* spp. are recognized for their diverse biocontrol mechanisms, including the secretion of secondary metabolites like pyrrolnitrin, phenazines, 2,4- diacetyl phloroglucinol (DAPG), and antifungal enzymes such as proteases, chitinases, and lipases^29^. Both strains, T17-4 and VUPf5, produced proteases, siderophores (not shown), and phenazine derivatives. Phenazine treatment inhibited the growth of Botrytis *cinerea*, resulting in altered fungal mycelia structure, reduced exopolysaccharide production, disrupted virulence factors, and hyphal network destruction^30^. Phenazine-1-carboxamide also demonstrated inhibitory effects on mycelial growth, virulence, and mycotoxin production in *F. graminearum* by affecting the fungal protein FgGcn5^31^. PCA (phenazine-1-carboxylic acid) was reported to suppress mycelial growth effectively^32^.

*B. velezensis* strains Q12 and US1 exhibited significant inhibition of *F. solani* both in laboratory and greenhouse conditions. In the presence of *B. velezensis* Q12, hyphal growth completely ceased, and sporulation was reduced. In contrast, B. *velezensis* US1 only halted mycelial growth, while the population of fungal spores remained unaffected. Furthermore, *B*. *velezensis* UR1 proved ineffective in controlling *F*. *solani*, as fungal filaments and spores proliferated and covered the bacterial cells. In the context of *F. solani*, each spore in the soil represents a pathogenic unit, and a higher number of pathogenic units contributes to increased disease prevalence in the soil^33^. Inhibiting pathogen growth and sporulation are critical for effective disease control, but the effectiveness of each strain may vary. Notably, *B*. *velezensis* Q12 exhibited distinct behavior from other strains within the same species. Volatile compounds released by *B. amyloliquefaciens* NJN-6 were reported to inhibit the growth and germination of *F. oxysporum* f. sp*. cubense* spores^34^. Iturin A, produced by *B. subtilis* WL-2, disrupts cell structure, induces oxidative stress, and disrupts energy supply in *P. infestans*^35^.

### Biocontrol of bacterial pathogens

Despite its effectiveness against fungi and bacteria, strain T17-4 exhibited signs of severe rot on potato tubers. Previous studies have shown that biofilms of *P. aeruginosa* PA14 produce R-body protein polymers that contribute to host colonization and pathogenicity^36^.

*Bacillus* fengycin suppresses Staphylococcus *aureus* by disrupting its quorum-sensing system^37^. A functioning quorum sensing system is essential for the pathogenicity of *Pectobacterium* spp. on potato tubers^38^, and Bacilli produce enzymes like lactonase that can disrupt the quorum sensing system activity in *Pectobacterium* spp.^39,40^. *B. velezensis* FZB42 harbors 11 gene clusters responsible for synthesizing antimicrobial metabolites^41^. Iturin and fengycin possess antifungal properties, while surfactin exhibits broad-spectrum antibacterial activity^42^. Additionally, *B*. *velezensis* bacilysin can suppress Gram-negative bacterial pathogens^43^. Overproduction of bacilysin enhances antagonistic effects against *Staphylococcus aureus* and *Clavibacter michiganense* subsp. *sepedonicum*^44^. Notably, strain Q12 effectively controlled all three bacterial pathogens compared to US1, although it did not prevent the growth of *P. carotovorum*. Strain Q12 was found to possess the locillomycin gene cluster, which encodes cyclic lipononapeptides with potent antibacterial activity^45,46^. Strain US1, on the other hand, contained the plantazolicin gene, which acts as an antibacterial agent against closely related Gram-positive bacteria, especially *B. anthracis*, the causative agent of anthrax^47,48^. PVDs (pyoverdines) in *P. chlororaphis* YL-1 are essential for a wide range of antibacterial activities against both Gram-positive and Gram-negative bacteria under low-iron conditions^49^. However, VUPf5 exhibited limited efficacy against bacterial pathogens compared to T17-4, which demonstrated excellent antibacterial activity against three Gram-negative bacteria. Previous studies have reported inhibitory activity against a wide range of Gram-negative bacteria, even in strains deficient in PCA production^50–54^. The *P. aeruginosa* PCA showed no effect against bacteria. An organocopper antibiotic compound (OAC) displayed potent antibiotic activity against *X. citri* subsp. *citri*. Electron microscopy analysis demonstrated that F3d affected exopolysaccharide production and caused cell lysis of the pathogen within the citrus canker lesions^55^.

Strain UR1 was found to produce no-traceable amounts of surfactin, fengycin, and iturin and was incapable of inhibiting *F. solani*, *Pectobacterium* spp., and *X. campestris*. Thus, these secondary metabolites play an essential role in controlling plant diseases both in vitro and in vivo. Previous studies have shown that the genes *codY*, *comA*, *degU*, and *spo0A* positively or negatively regulate the biosynthesis of bacillomycin D, fengycin, and surfactin in *B. amyloliquefaciens* fmbJ^56^. It has also been found that the expression of the *sfp* gene is essential for the biosynthesis of lipopeptides and polyketides in *B. subtilis* and *B. velezensis* FZB42^14^. In *B. subtilis* 3610, five genes (*sfp*, *degQ*, *epsC*, *swrA*, and *spo0F*) are located on a large plasmid^14,57^. Studies suggest that surfactin and bacillomycin in *B. subtilis* 916 synergistically control *Rhizoctonia solani* in rice through their antifungal action^58^.

### Biocontrol of *F. solani* in the potato rhizosphere

Among the *B. velezensis* strains tested, Q12 demonstrated the most significant improvement in total plant and root weight. Plant growth-promoting rhizobacteria like *B. velezensis* trigger mechanisms for the biocontrol of plant pathogens, including direct suppression through secondary metabolites, induced systemic resistance (ISR), and competitive colonization of the plant rhizosphere^16^. Lipopeptides, for example, play a role in ISR, swarming, biofilm formation, and colony morphology improvement^59^. Iturin has been reported to induce MAMP-triggered immunity defense in cotton plants, leading to ROS burst, disruption of cell-wall integrity, and interference with fungal signaling pathways^60^.

### Genetic findings support Q12 as an outstanding biological control bacteria and fertilizer among 500 bacterial isolates and two positive biocontrol bacteria

In our previous screening of 900 strains, we identified VUPf5 as a potent controller of fungal diseases but with limitations in controlling bacterial diseases. To identify more powerful strains, VUPf5 was tested alongside other strains. Strain T17-4, apart from causing potato tuber rot, is known to be a human pathogen. Therefore, confirming its safety for human health is crucial (Fig. S5, Supplementary Table 1). After identifying human pathogenic strains, we meticulously implemented laboratory safety protocols to ensure proper containment.

We employed various servers to analyze and compare the genetic profiles of Q12 and US1. While the DAVID server provided a general comparison of the strains, RAST and antiSMASH offered more comprehensive insights into their functional differences. Annotation of the Q12 genome revealed an additional 45 coding genes and 49,573 bps of non-coding sequences compared to the US1 genome.

Parallel studies have consistently shown that Q12 outperforms other strains. For example, Q12 exhibited robust colonization of tomato roots, forming a strong biofilm in both in vitro and in vivo settings, as observed through Confocal Laser Scanning Microscopy (CLSM) analysis. Furthermore, Q12 demonstrated higher tolerance to high salt concentrations (10% NaCl) and distinguished itself from the other seven strains, including US1, in these experiments^61^. Q12 possesses additional genes in various categories, particularly those related to the cell wall, capsule, stress response (oxidative stress and sigmaB stress response regulation), polysaccharide metabolism, sucrose metabolism, and levansucrase synthesis (EC 2.4.1.10). It also harbors a complete gene cluster for locillomycin polypeptide. Levan, an exoenzyme levansucrase, contributes to salt tolerance and facilitates the formation of robust biofilms, which, in turn, protect bacteria from biotic and abiotic stresses such as dryness, salinity, and high sucrose concentrations^62,63^.

Under salt stress conditions, *Bacillus* sp. bacteria upregulate the expression of *sacB*, encoding levansucrase, and the two-component system DegS-DegU to sense salt stress^64^. In many Bacillales members, the sigma factor B (σB or SigB) regulates the transcription of general response genes in response to environmental and nutritional stresses, including ethanol, salt, glucose starvation, heat, and cold. This activation leads to the expression of SigB-related genes and proteins that protect cells from damage, as observed in Q12^65–68^. These genes, along with others identified in Q12, likely contribute to osmotic stress resistance and promote biofilm formation, enhancing root colonization.

Although both Q12 and US1 possess four genes related to auxin biosynthesis, significant differences in potato plant growth were evident in the presence of Q12. Furthermore, the Q12 genome features a gene associated with polysaccharide metabolism, potentially contributing to its ability to utilize diverse food sources and effectively compete and colonize roots in the rhizosphere. Polysaccharides secreted by plant roots serve as a vital nutrient source for surrounding microbes and act as cues for biofilm formation and root colonization by *B. subtilis*^69^.

In this study, Q12 demonstrated a significant advantage in controlling both bacterial and fungal pathogens and promoting plant growth in laboratory and greenhouse settings compared to other strains. These phenotypic and functional advantages can be attributed to the strain's superior genetics, as indicated by the presence of valuable genes not found in US1. Consequently, we introduce Q12 as an exceptional strain among the 500 tested, showcasing its potential for biological control of potato plant diseases and enhancement of growth rates.

## Conclusion

This study has demonstrated the efficacy of selected strains of *B. velezensis* (Q12 and US1) and our previously identified biocontrol strain *P. chlororaphis* VUPf5 in the biocontrol of fungal dry wilt in potatoes. Notably, VUPf5 exhibited potent control over fungal diseases, disrupting the mycelium network. However, it displayed limitations in controlling bacterial diseases. Meanwhile, *P. aeruginosa* T17-4, in addition to inducing rot in potato tubers, is recognized as a human pathogen, necessitating a thorough assessment of its safety for human health. Nevertheless, T17-4 displayed significant capabilities in controlling fungal and bacterial pathogens under laboratory conditions.

*B. velezensis* strains Q12 and US1 demonstrated effective inhibition of *F. solani* under both laboratory and greenhouse conditions. Notably, strain Q12 exhibited exceptional capabilities in controlling fungal pathogens and addressing bacterial soft rot in potatoes, ultimately promoting enhanced plant growth compared to other strains. These strains employ diverse biocontrol mechanisms, encompassing the secretion of secondary metabolites such as siderophores, phenazine derivatives, iturin, surfactin, fengycin, and antifungal enzymes like proteases, chitinases, and lipase. The production of secondary metabolites such as iturin, fengycin, surfactin, and locillomycin appears to play a significant role in the biocontrol mechanisms of *B. velezensis* strains. Notably, these lipopeptides were not secreted in *B. velezensis* UR1, highlighting its distinct behavior within the same species. Furthermore, the study underscores the importance of controlling pathogen growth and sporulation in the management of plant diseases, with variations in effectiveness among different species. Genome mining analyses revealed that strain Q12 possesses additional coding and non-coding sequences compared to strain US1, potentially contributing to its biocontrol efficacy. The presence of the locillomycin synthetizing gene cluster in strain Q12 holds promise for controlling bacterial soft rot in potatoes (*Pectobacterium* spp.) and *Xanthomonas campestris*, although further comprehensive exploration is warranted.

Future research endeavors may explore the potential synergistic effects of combining different strains of *B. velezensis* and *P. chlororaphis* VUPf5 to enhance the biocontrol of plant diseases. Moreover, these findings have the potential to underpin the development of sustainable strategies for plant disease management in agriculture. The utilization of biocontrol agents such as *B. velezensis* and *P. chlororaphis* could mitigate the reliance on chemical pesticides, known for their adverse environmental and health impacts. Furthermore, insights gained from this study regarding the biocontrol mechanisms employed by these bacterial species may inform the development of novel biopesticides and biostimulants. In summary, this study underscores the promise of selected strains of *B. velezensis* and *P. chlororaphis* VUPf5 for the biocontrol of plant diseases, with the prospect of further optimizing their utilization in sustainable agricultural practices.

### Supplementary Information

Supplementary Legends.

Supplementary Figure S1.

Supplementary Figure S2.

Supplementary Figure S3.

Supplementary Figure S4.

Supplementary Figure S5.

Supplementary Tables.

## Data Availability

The whole genome sequencing data were deposited in the NCBI database with the Accession Numbers CP076290-CP076292, and the sequences data with the Accession Numbers MZ357346-MZ357348, MZ310706, MZ310808, MZ310821, MZ346009, MZ349056, MZ346008, MZ312100 and MZ310814. The mass spectrometry data are available from the corresponding author upon reasonable request.
