# Supplementary material for: Genome mining conformance to metabolite profile of Bacillus strains to control potato pathogens
Source: Sci Rep. 2023 Nov 4;13:19095. doi: 10.1038/s41598-023-46672-1 (PMC10625545; doi:10.1038/s41598-023-46672-1)
Supplement: Supplementary file 1 — Supplementary Legends. [file 41598_2023_46672_MOESM1_ESM.pdf]

Supplementary legends

Fig. S1 a-d. Interaction between biocontrol bacteria and pathogenic fungus *F. solani*, by stereomicroscope. The end margin of fungal mycelia and spore growth (fungal organelles) in the vicinity of bacteria. **a**. Positive Control (without biocontrol bacteria). **b**. *B. velezensis* Q12. **c**. *B. velezensis* US1. **d**. *P. chlororaphis* VUPf5.

Fig. S2. Interaction between biocontrol bacteria and pathogenic fungus *F. solani*, by stereomicroscope. Arrows indicate fungal organelles in the vicinity of bacteria. **a**. Exit and growth of germ tube from fungal spores next to UR1. **b**. Creating a large number of phialides in treatment with UR1 (Defects in the production of iturin, fengycin, and surfactin). **c**. Exit and growth of germ tube from fungal spores next to *E. coli* OB63

Fig. S3. Molecular identification of bacterial strains. Size Marker (1 Kb plus, Thermo Fisher). Amplified by 16sRNA primers (27F and 1492R), *gyrA* primers, and *rpoD* primers (left to right).

Fig. S4. Investigating the production of secondary metabolites in *B. velezensis* UR1 using LC/MS/MS

Fig. S5 a-c. Investigating the effect of biological control of *Pectobacterium carotovorum* on the potato tuber. **a**. *B. velezensis* Q12. **b**. *P. aeruginosa* T17-4. **c**. Positive Control (only *P. carotovorum)*
