# Supplementary figures and images for: Genome mining conformance to metabolite profile of Bacillus strains to control potato pathogens
Source: Sci Rep. 2023 Nov 4;13:19095. doi: 10.1038/s41598-023-46672-1 (PMC10625545; doi:10.1038/s41598-023-46672-1)

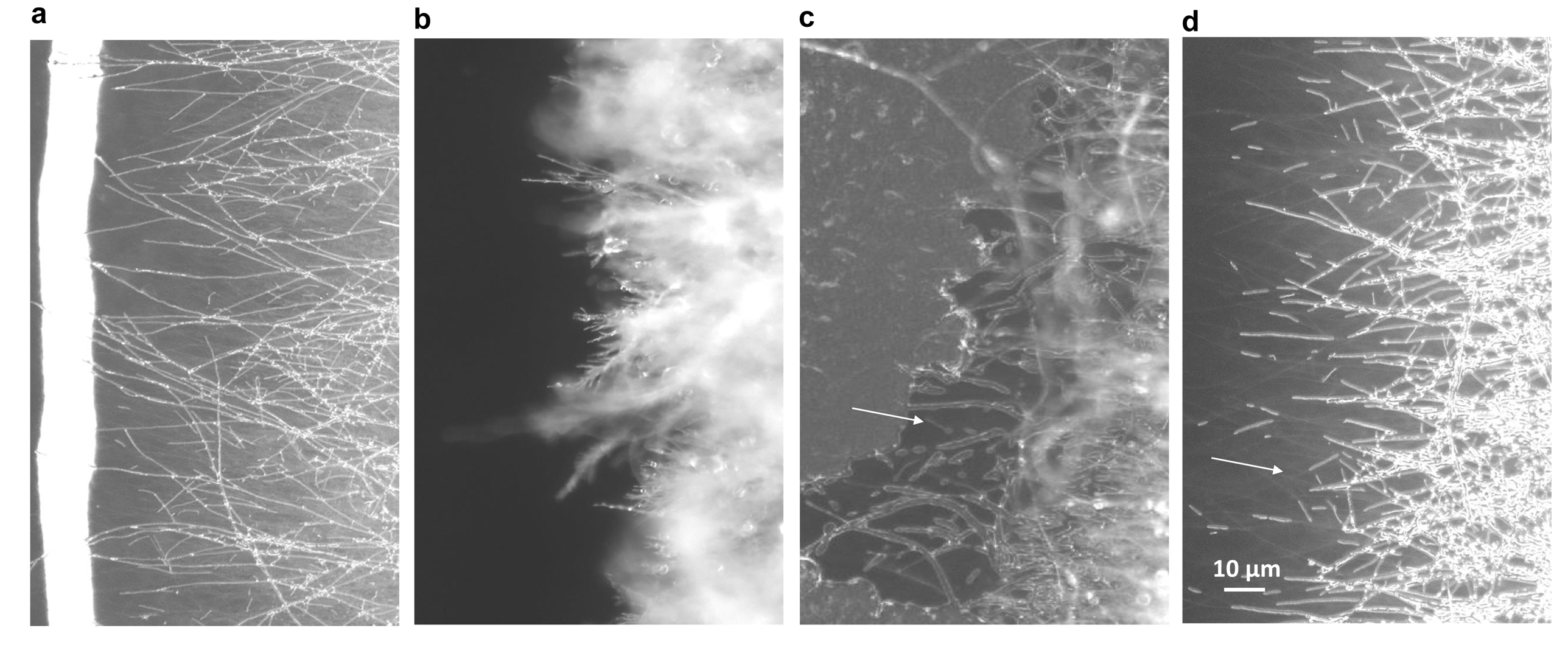

Supplement: Supplementary file 2 — Supplementary Figure S1. [file 41598_2023_46672_MOESM2_ESM.tif]

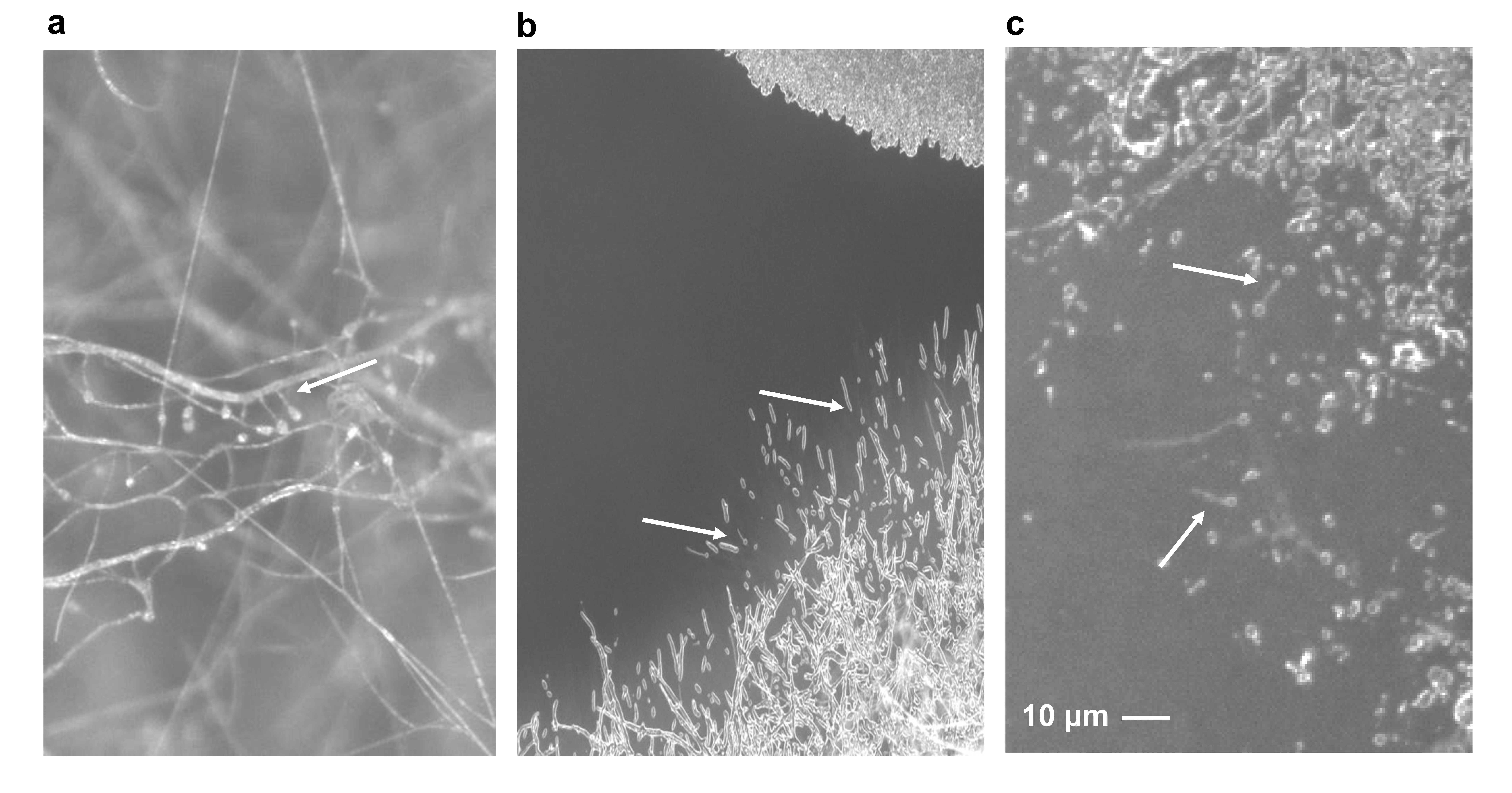

Supplement: Supplementary file 3 — Supplementary Figure S2. [file 41598_2023_46672_MOESM3_ESM.tif]

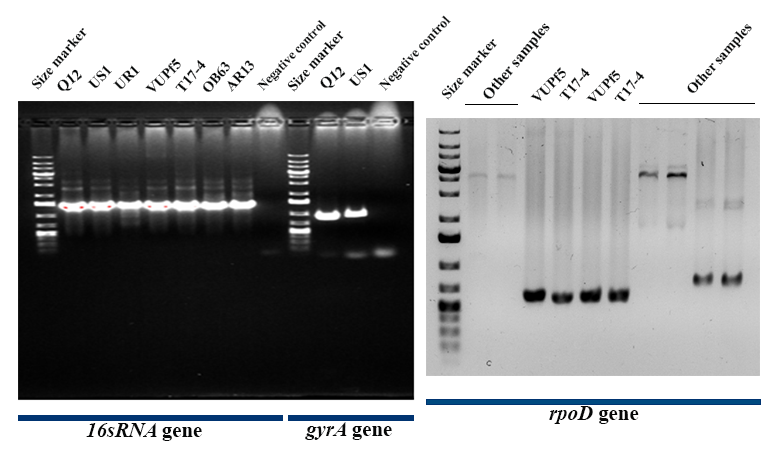

Supplement: Supplementary file 4 — Supplementary Figure S3. [file 41598_2023_46672_MOESM4_ESM.tif]

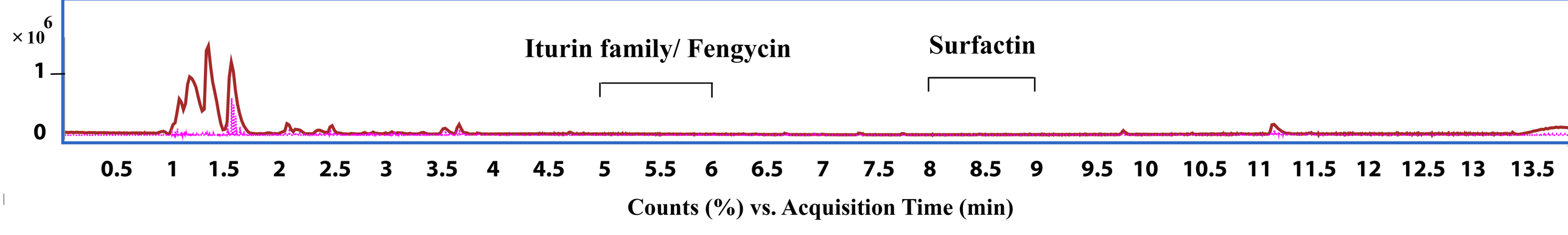

Supplement: Supplementary file 5 — Supplementary Figure S4. [file 41598_2023_46672_MOESM5_ESM.tif]

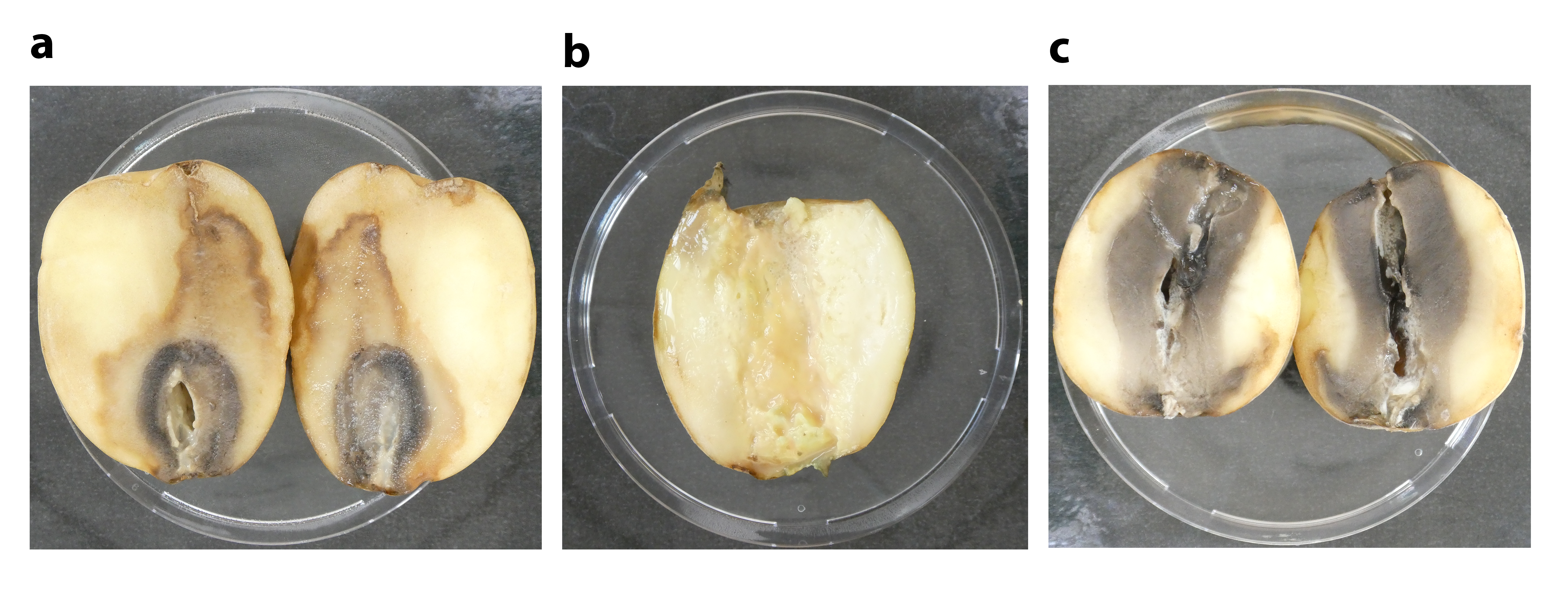

Supplement: Supplementary file 6 — Supplementary Figure S5. [file 41598_2023_46672_MOESM6_ESM.tif]
