# Supplementary material for: Genome mining conformance to metabolite profile of Bacillus strains to control potato pathogens
Source: Sci Rep. 2023 Nov 4;13:19095. doi: 10.1038/s41598-023-46672-1 (PMC10625545; doi:10.1038/s41598-023-46672-1)
Supplement: Supplementary file 7 — Supplementary Tables. [file 41598_2023_46672_MOESM7_ESM.pdf]

Supplementary Table 1a. Inhibition rate of pathogen growth on the plated media

**Biocontrol bacteria**	**Inhibition rate**			
	***F. solani* (%)**	***P. carotovorum* (mm)**	***P. atrosepticum* (mm)**	***X. campestris* (mm)**
B. velezensis Q12	48 a	0.12 b	0.21 b	0.51 b
B. velezensis US1	48 a	0.06 c	0.15 c	0.3 c
B. velezensis UR1	23 def	0 e	0 g	0 g
P. chlororaphis VUPf5	38 b	0 e	0 g	0.29 d
P. aeruginosa T17-4	35 bc	1.64 a	0.89 a	1.1 a
E. coli OB63	29 cd	0 e	0 g	0 g
E. coli AR13	24 de	0 e	0 g	0.01 f
M44	20 efg	0 e	0 g	0 g
AR8	19 efgh	0 e	0 g	0 g
E15	17.7 efgh	0.042 d	0.052 d	0.08 e
H10	18.4 efgh	0 e	0 g	0 g
AR3	18.4 efgh	0.01 e	2 e	0.01 f
Q5	12.33 h	0 e	0 g	0 g
M17	17.2 efgh	0 e	0.011 f	0 g
H5	16.3 fgh	0.01 e	0 g	0 g
OB59	16 gh	0 e	0 g	0 g
E8	16.3 fgh	0.05 cd	0 g	0 g
E3	16.2 fgh	0 e	0 g	0 g
E11	15.3 gh	0 g	0 g	0 g
J9	15.3 gh	0 g	0 g	0 g

Statistical analysis of each column was done separately with the One-Way ANOVA, Post-hoc tests (Tukey test) at a significant level of 0.05. Post-hoc tests indicated significant differences between the treatments in the control groups of *F. solani* (F= 68.04, df= 19, P= 0.000), *P. carotovorum* (F= 26062.91, df=19, P= 0.000), *P. atrosepticum* (F= 1220653.50, df=19, P= 0.000), and *X. campestris* (F= 180925.55, df= 19, P= 0.000).

Supplementary Table 1b. Inhibition percentage of pathogen growth *in vivo*

**Biocontrol bacteria**	**Inhibition percentage**		
	***F. solani*[1]**	***P. carotovorum*[*]**	***P. atrosepticum*[*]**
B. velezensis Q12	100 a	50 a	52.7 a
B. velezensis US1	85 c	8.3 b	10.1 b
B. velezensis UR1	45 f	0 d	0 d
P. chlororaphis VUPf5	90 b	5.2 c	8.5 c
P. aeruginosa T17-4	90 b	0 d	0 d
E. coli OB63	50 e	0 d	0 d
E. coli AR13	55d	0 d	0.03 d
E11	0 g	0 d	0 d
J9	0 g	0 d	0 d

[1] The experiment was carried out under greenhouse conditions. [*] The control of pathogens was checked on potato tubers. [1,*] Statistical analysis of each column was done separately with the One-Way ANOVA, Tukey test at a significant level of 0.05. Post-hoc tests showed significant differences between the treatments in the groups of *F. solani* (F= 31063.40, df= 8, P= 0.000), *P. carotovorum* (F= 9666.00, df= 8, P= 0.000), and *P. atrosepticum* (F= 3408.36, df= 8, P= 0.000).

Supplementary Table 1c. PGPR characteristics of biocontrol bacteria in greenhouse conditions

Biocontrol bacteria	Growth rate (gr)	
	Total plant weight*	Root weight*
B. velezensis Q12	54.77 a	7.6 a
B. velezensis US1	44 b	4.93 b
B. velezensis UR1	29.33 e	3.1 e
P. chlororaphis VUPf5	45.13 c	5 c
P. aeruginosa T17-4	44.43 c	5.1 c
E. coli OB63	26.2 f	2.53 f
E. coli AR13	26 f	2.44 f
E11	24 g	2.7 g
J9	23.83 g	2.65 g
FS	21.48 h	2.5 g
CN	35.2 d	3.67 d

* Statistical analysis of each column was done separately with the One-Way ANOVA, Duncan test at a significant level of 0.01. The analysis showed significant differences between the treatments in two groups of total plant weight (F= 1642.26, df= 10, p= 0.001) and root weight (F= 713.78, df= 10, p= 0.001). FS: The treatment of the infected with *F. solani*. CN: The treatment without pathogen contamination and biocontrol bacteria.

Supplementary Table 1d. Detected biocontrol secondary metabolites using whole genome sequencing and ultrahigh-performance liquid chromatography; high-resolution electrospray ionization mass spectrometry.

Biocontrol bacteria	Biocontrol secondary metabolite
B. velezensis Q12	iturin A- fengycin- surfactin- locillomycin- butirosin A/B- macrolactin H- bacillaene- difficidin- bacillibactin- bacilysin
B. velezensis US1	bacillomycin D -fengycin - surfactin -plantazolicin- butirosin A/B- macrolactin H- bacillaene- difficidin- bacillibactin- bacilysin
P. chlororaphis VUPf5	phenazine-1-carboxylic acid- 2-hydroxyphenazine-1-carboxylic acid- 2-hydroxyphenazine* - lahorenoic acids A
